# Hierarchical and automated cell-type annotation and inference of cancer cell of origin with Census

**DOI:** 10.1093/bioinformatics/btad714

**Published:** 2023-11-27

**Authors:** Bassel Ghaddar, Subhajyoti De

**Affiliations:** Center for Systems and Computational Biology, Rutgers Cancer Institute of New Jersey, Rutgers University, New Brunswick, NJ 08901, United States; Center for Systems and Computational Biology, Rutgers Cancer Institute of New Jersey, Rutgers University, New Brunswick, NJ 08901, United States

## Abstract

**Motivation:**

Cell-type annotation is a time-consuming yet critical first step in the analysis of single-cell RNA-seq data, especially when multiple similar cell subtypes with overlapping marker genes are present. Existing automated annotation methods have a number of limitations, including requiring large reference datasets, high computation time, shallow annotation resolution, and difficulty in identifying cancer cells or their most likely cell of origin.

**Results:**

We developed Census, a biologically intuitive and fully automated cell-type identification method for single-cell RNA-seq data that can deeply annotate normal cells in mammalian tissues and identify malignant cells and their likely cell of origin. Motivated by the inherently stratified developmental programs of cellular differentiation, Census infers hierarchical cell-type relationships and uses gradient-boosted \decision trees that capitalize on nodal cell-type relationships to achieve high prediction speed and accuracy. When benchmarked on 44 atlas-scale normal and cancer, human and mouse tissues, Census significantly outperforms state-of-the-art methods across multiple metrics and naturally predicts the cell-of-origin of different cancers. Census is pretrained on the Tabula Sapiens to classify 175 cell-types from 24 organs; however, users can seamlessly train their own models for customized applications.

**Availability and implementation:**

Census is available at Zenodo https://zenodo.org/records/7017103 and on our Github https://github.com/sjdlabgroup/Census.

## 1 Introduction

Single cell RNA-seq (scRNA-seq) has enabled annotation and transcriptional characterization of cell-types in multicellular species. Cell-type annotation is a critical and often difficult and time-consuming first step in any scRNA-seq data analysis. Typical annotation pipelines involve cell clustering followed by comparison of cluster differentially expressed genes with cell-type marker genes databases (Clarke *et al.* 2021). While this approach is suitable for major, well-defined cell-types, it can be challenging to annotate cells from noisy datasets or to identify cell-subtypes for which marker genes are overlapping, poorly expressed, or incompletely described (Shekhar and Menon 2019, Lähnemann *et al.* 2020). This problem is especially pertinent while analyzing scRNA-seq data from perturbation experiments, disease contexts such as cancer, or treatment conditions.

As such, a number of automated cell identification methods have been developed (Aran *et al.* 2019, de Kanter *et al.* 2019, Zhang *et al.* 2019, Li *et al.* 2020, Shao *et al.* 2020, Cortal *et al.* 2021, Guo and Li 2021, Ianevski *et al.* 2022, Nofech-Mozes *et al.* 2022, Zhang *et al.* 2022), and cell types in many organ-types have been annotated (Schaum *et al.* 2018, Jones *et al.* 2022). However, when applied to complex tissues, in practice, multiple limitations of these approaches become apparent (Abdelaal *et al.* 2019, Pasquini *et al.* 2021, Xie *et al.* 2021). These include inaccurate or shallow annotations, limited organ or cell-type scope, long computation time, the requirement of large reference data, or an inability to distinguish between malignant cells and their normal counterparts (Abdelaal *et al.* 2019, Pasquini *et al.* 2021, Xie *et al.* 2021). In addition, batch effects or differences in cell subtypes between reference and test data often lead to incorrect label predictions or resolutions. Without clearly defined hierarchical cell-type relationships, it can be difficult to identify the appropriate cell ontological resolution or alternative cell-type annotations. To overcome the current challenges in scRNA-seq cell-type annotation, we developed Census, a fast and fully automated hierarchical cell-type identification method that is conceptually motivated by inherently stratified developmental programs of cellular differentiation.

## 2 Materials and methods

### 2.1 Census algorithm

**Figure 1.**
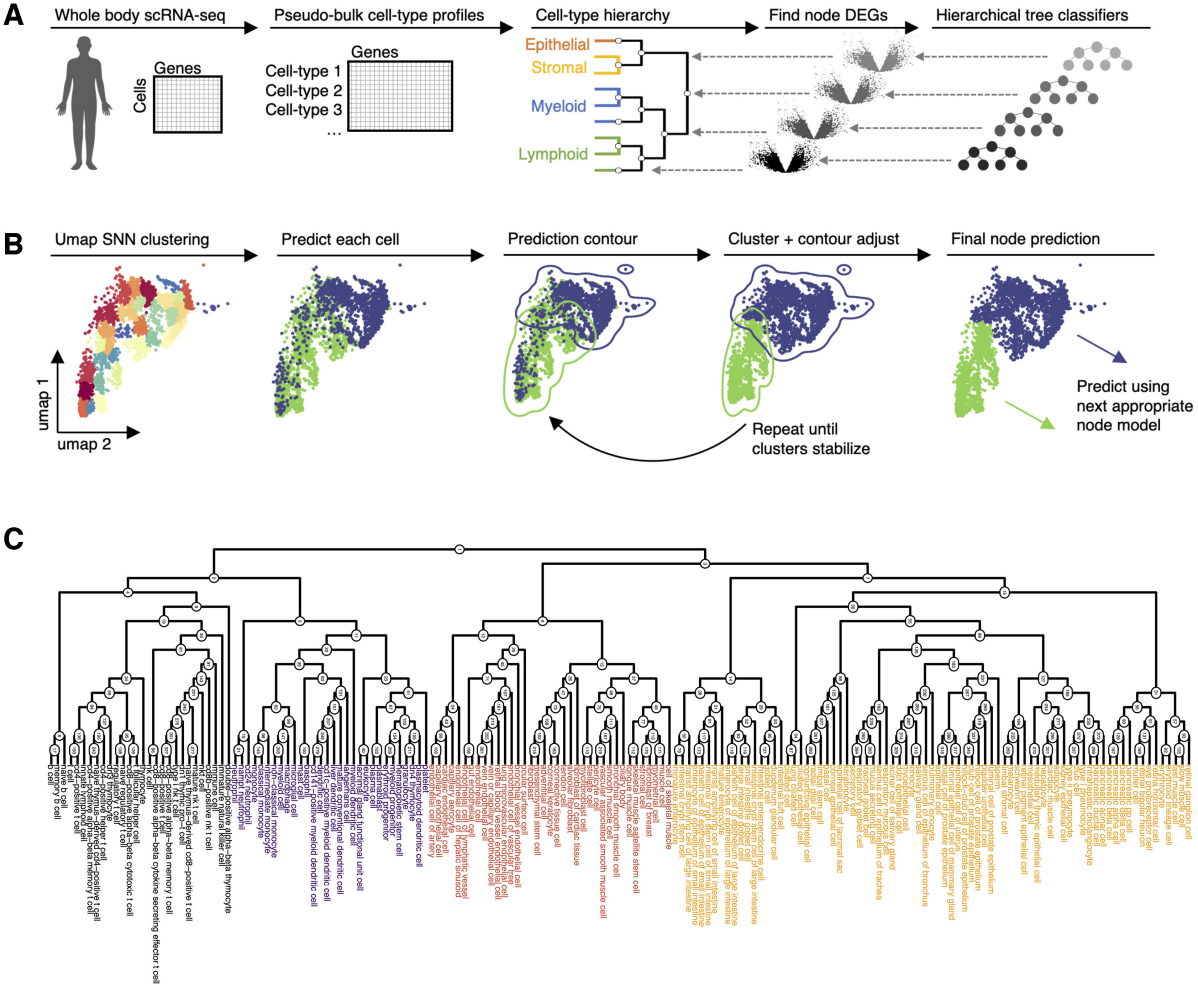
Overview of Census and the Tabula Sapiens cell-type hierarchy. (**A**) Schematic diagram of training a Census model. The default Census model is trained on the Tabula Sapiens and Cancer Cell Line Encyclopedia. Given a reference dataset, Census computes a cell-type hierarchy using pseudo-bulk cell-type profile, identifies differentially expressed genes at each node of the hierarchy, and trains a classifier model for each node. (**B**) Schematic diagram of the Census prediction method and label-stabilizing algorithm. Query cell identities are successively predicted using the hierarchical model. Prediction noise is mitigated using a prediction contour adjustment algorithm. (**C**) Dendrogram plotting the cell-type hierarchy derived from the Tabula Sapiens. The major cell compartments that emerge lymphoid, myeloid; stromal, and epithelial.

*Constructing the cell-type hierarchy:* Census begins by constructing a cell-type hierarchy from reference scRNA-seq data (Fig. 1A). Given all gene expression and cell-type labels, pseudo-bulk cell-type profiles are created by summing gene counts across all barcodes per cell-type, creating a gene by cell-type table. The resulting profiles are TP10K normalized and then hierarchically clustered using Ward’s method, which clusters each node into two leaves. Each node of the hierarchical tree is numbered, and the terminal leaves represent the final cell-types.

*Training a Census model:* A collection of gradient-boosted tree-based classification models (Chen and Guestrin 2016) organized by the cell-type hierarchy are next trained to predict cell-type label from scRNA-seq gene expression values. Each node of the cell-type hierarchy has an associated classification model; there are as many models as there are nodes in the cell-type hierarchy. Starting with the root node of the cell-type hierarchy, the cells of all downstream cell-types whose lineage contains the given node are gathered. All nodes bifurcate into two child nodes; the task of the node model of the given node is to classify cells into the appropriate child node of the given node. Cells from the training data are thus given the new identity of their respective child node of the given node through which their lineage runs. This results in two identity classes, and marker genes that distinguish these two classes are identified using Wilcoxon Rank Sum testing, as implemented in Seurat (Stuart *et al.* 2019). By default, all statistically significant marker genes are used (i.e. adjusted Wilcoxon rank sum test *P*-value < 0.05); this usually results in a sufficiently large number of genes, although users may impose custom filters or provide alternate marker gene data for the models to use. The node model uses marker gene counts data to predict the associated cell-label.

Census modifies the training data in three ways before model training. First, zero-values are replaced with NA (not available) to be treated as missing values by the classification algorithm. This is done to account for variable dropout levels across scRNA-seq datasets and across individual cells in a given dataset, wherein zero-values may represent lack of detectable gene expression, low gene expression confounded by measurement noise, or uncaptured gene expression. This also accounts for potentially missing genes between the training data and test datasets, and it takes advantage of the underlying sparsity-aware split finding algorithm developed explicitly by xgboost (Chen and Guestrin 2016) to optimize handling of missing values in classification problems. Second, gene values for each cell are percentile ranked. This helps mitigate batch effects across studies and effects of varying sequencing depth; it forces the model to focus on relative ordering or genes instead of exact values. Third, Census supplements the training data with a sparsified dataset; this is done by replacing a default of 90% (users may control this from 0% to 99%) of the gene counts with missing values, and gene counts are then percentile ranked. The addition of sparsified data was found to improve model performance across a range of datasets that had very different sequencing depths. The full and sparse training data are combined, and a classification model is trained to predict the cell-label given the gene expression data. This process is repeated for each node of the cell-type hierarchy, with the final models predicting terminal cell-type labels. The above design choices make Census robust to missing values and batch effects.

*Annotating cell-types in new datasets:* Census uses the resulting models in conjunction with a custom label-stabilizing algorithm to predict new datasets (Fig. 1B). First, the test dataset is processed using standard scRNA-seq pipelines to project it in two dimensions using uniform manifold approximation and projection (UMAP, i.e. by TP10K normalization, scaling, finding variable genes, computing principle components, and then running the UMAP algorithm using the top principle components), as implemented in Seurat (Stuart *et al.* 2019). This data is finely clustered using the first two UMAP dimensions using a shared nearest-neighbor (SNN) algorithm, as implemented in Seurat (Stuart *et al.* 2019). These clusters represent groups of highly similar cells in the test dataset and are used to mitigate prediction error in individual cells. It is crucial at this step that high resolution clustering is done to take advantage of UMAP’s preservation of local structure and to avoid co-clustering distant cells.

Next, starting with the first model corresponding to the root node of the cell-type hierarchy, new cell identities are predicted for each individual cell in the test dataset. Census then implements a custom label-stabilizing algorithm that counteracts potential dataset noise and prediction error. First, the average label is propagated within each UMAP SNN cluster. Next, prediction contours are computed on the UMAP plot using the MASS (Veneables and Ripley 2002) R package. In areas where prediction contours do not overlap, all cells within the contour are given the identity of the contour. In areas where the prediction contours overlap, cells within the overlapping region are given the identity of the most common label in that region. After resolving contour disputes, the most common label is again propagated across each UMAP SNN cluster, and new prediction contours are computed. This process is repeated until either there are no more overlapping prediction contours or until there are no further changes to any cell labels. Each cell now has a new identity, and the next appropriate node model is used to predict subsequent labels; this process is repeated until terminal cell-type classifications are reached. A record of predicted classes and probabilities for each cell in each round of classification is retained.

### 2.2 Census models

The core census model was trained on the Tabula Sapiens (Jones *et al.* 2022) to classify 175 cell-types from 24 organs (Fig. 1C). The cell-type hierarchy contained 345 nodes, with the first node bifurcating into immune versus nonimmune cells, and then further branches dividing into B-lymphoid, T-lymphoid, myeloid, endothelial, stromal, and epithelial compartments. The Tabula Sapiens was chosen as the reference for the core model due to its comprehensive human body profiling and consistent cell class ontology labeling, and through extensive benchmarking experiments the core model was found to generalize well across a range of datasets, although users can train their own annotation models.

To identify and annotate cancer cells, we trained models on scRNA-seq data from 22 cancer types from the Cancer Cell Line Encyclopedia (Kinker *et al.* 2020) to distinguish malignant cells from organ-specific normal epithelium. For example, to identify cancer cells in the pancreas, the classification model was trained to distinguish between the cancer cell line data and pancreas epithelium from the Tabula Sapiens, i.e. ductal, acinar, and endocrine cells. Users may also consider training the models using scRNAseq data from tumors [e.g. Human Tumor Atlas Network (Rozenblatt-Rosen *et al.* 2020)], as relevant data becomes available. When predicting new datasets, the Census model begins by finding terminal classifications for all cell-types using the Tabula Sapiens trained model and cell-type hierarchy. If cancer cells are expected in the sample, then the organ-specific cancer model is applied only to the cells classified as epithelial cells by the Tabula Sapiens model to identify cancer cells. The same contour and cluster-based label stabilizing algorithm is applied. The final output will contain cell-type predictions, and for the predicted cancer cells, and it will also retain the origin normal cell-type prediction as the predicted cell of origin. Cancer cell type models are available for the following organs: breast, colon, kidney, liver, lung, and pancreas. While the Tabula Sapiens and cancer models enable rapid and automated cell-type identification for a variety of datasets, users can also easily train new models with other references (which may include cancer cells as part of the reference) for custom applications.

### 2.3 Benchmarking analyses

All benchmarking analyses were conducted using new datasets not used in training and testing the Tabula Sapiens Census model. Initial benchmarking compared Census to four other state-of-the-art automated annotation methods: scType (Ianevski *et al.* 2022), scATOMIC (Nofech-Mozes *et al.* 2022), scibet (Li *et al.* 2020), and scCATCH (Shao *et al.* 2020). For scType, the primary tissue type as well as “immune system” were chosen for as the tissue type. For scibet, the “30_major_human_cell_types” model was used. Other methods were run with default parameters. All methods were evaluated using on a pancreatic cancer dataset (Peng *et al.* 2019). Census and scType were further evaluated on two datasets each of colon (Lee *et al.* 2020, Pelka *et al.* 2021), kidney (Bi *et al.* 2021, Krishna *et al.* 2021), liver (Massalha *et al.* 2020, Ma *et al.* 2021), lung (Kim *et al.* 2020, Wu *et al.* 2021a), and pancreas (Peng *et al.* 2019, Hwang *et al.* 2022) cancers, normal lung (Travaglini *et al.* 2020), colon (Smillie *et al.* 2019), and heart (Litviňuková *et al.* 2020) datasets, and tissues from the Tabula Muris (Schaum *et al.* 2018) where applicable. Census was additionally evaluated on two datasets of human breast cancer (Wu *et al.* 2020, Wu *et al.* 2021b). In total, Census was evaluated on 44 tissue samples from 23 unique tissue types that contained 1 769 071 total cells from 105 harmonized cell labels.

To assess performance, original author labels had to be harmonized with the cell ontology annotations used in the Tabula Sapiens (Jones *et al.* 2022). This was done manually by relabeling each annotation with either the exact match in the Tabula Sapiens or the closest matching label. In some cases, the original author annotations were more deeply annotated than the Tabula Sapiens ontology (e.g. germinal center B cell, or inflammatory monocyte). In such cases the closest lower resolution label was chosen (e.g. B cell, monocyte). The closest appropriate label was decided by the authors on a case-by-case basis based on an understanding of the author annotated and Tabula Sapiens cell-type annotations.

Once labels were harmonized, F1 scores and balanced accuracies were calculated using the caret R package (https://topepo.github.io/caret/index.html). Label similarity scores to assess closeness of a predicted label to the original author annotated label were calculated as follows. First, cell-type labels from the original studies and the predicted labels from scType (Ianevski *et al.* 2022), scATOMIC (Nofech-Mozes *et al.* 2022), scibet (Li *et al.* 2020), and scCATCH (Shao *et al.* 2020) were harmonized to the cell ontology annotations used in the Tabula Sapiens (Jones *et al.* 2022) using the closest matching label. Then using the cell-type hierarchy created from the Tabula Sapiens, the label similarity score was calculated for each cell-type prediction as the percent of shared nodes of the shorter of the lineages of either the author annotated label or the predicted cell-label. Each individual cell thus had a label-similarity score, and each cell-type from each tissue sample had an F1 score and balanced accuracy. Wilcoxon Rank Sum tests were used to compare metrics for the different cell-type annotation methods.

### 2.4 Statistical analyses

All statistical analyses were performed using R version 3.6.1 (https://www.r-project.org/). The ggpubr package (https://github.com/kassambara/ggpubr) was used to compare group means with nonparametric tests. *P*-values reported as <2e−16 result from reaching the calculation limit for native R statistical test functions and indicate values below this number, not a range of values. Data processing relied heavily on the Tidyverse v1.3.2 R packages (https://www.tidyverse.org/).

## 3 Results

Census implements a collection of hierarchically organized gradient-boosted decision tree models (Chen and Guestrin 2016) that successively classify individual cells according to a predefined cell hierarchy (Fig. 1A). Briefly, Census begins by identifying a cell-type hierarchy from reference scRNA-seq data by hierarchically clustering pseudo-bulk cell-type gene expression data using Ward’s method, which splits each node into two child nodes. Next, starting with the root node and for each successive node, differentially expressed genes that distinguish cells from the two child nodes are identified and used as features to train a gradient-boosted tree model to classify the node identity of individual cells. Census uses multiple, relevant percentile-ranked feature scores, allows for missing values, and trains on both full and sparsely down-sampled data, resulting in models that are robust to batch effects.

New datasets are annotated using the pretrained models followed by a custom developed label-stabilizing algorithm (Fig. 1B). Census first uses uniform manifold approximation and projection (UMAP) and a SNN graph (Stuart *et al.* 2019) to finely cluster scRNAseq data. It begins by annotating cellular barcodes with the root classifier. Next, the average label per cluster is propagated and prediction contours in UMAP space are computed. Census resolves disputes within overlapping contour regions and repeatedly redraws contours until the prediction contours stabilize (see Methods for details). The label stabilizing algorithm thus uses two steps: first immediate cell neighborhoods defined by high-resolution UMAP clustering are given the same label, and second the prediction contour corrections leverage annotations across all clusters and adjusts potentially incorrectly labeled clusters if they are situated completely within by other clusters. Often only a single round of label stabilization is sufficient, however for noisy datasets this approach leads to improved classification performance and is computationally efficient, as will be demonstrated. Once this step is completed, each cellular barcode is assigned a new node identity, the next appropriate node classifier is applied, and this process is repeated until terminal classifications are reached. Census thus leverages multiple design features to achieve high speed and accuracy.

We trained Census using 175 cell-types from 24 organs using data from the Tabula Sapiens (Jones *et al.* 2022). Construction of the cell-type hierarchy revealed biologically meaningful groups, with the largest split being immune versus nonimmune cells and with cells further segregating into lymphoid, myeloid, endothelial, stromal, and epithelial groups (Fig. 1C). To identify and annotate cancer cells and distinguish them from the normal epithelial cells in the respective organs, we trained models on scRNA-seq data from 22 cancer types from the Cancer Cell Line Encyclopedia (Kinker *et al.* 2020). The total collection of models had 351 nodes, and all node models had high training classification accuracy (median AUC = 0.99). The Tabula Sapiens cell-type hierarchy Census identified shares many similarities to other cell ontologies (Chen *et al.* 2022) (Supplementary Fig. S1), namely broad grouping of cells from common progenitors. However, we note that Census does not aim to reconstruct ideal cell ontologies. Rather, the purpose of the cell-type hierarchy is to identify groups of cells with shared transcriptional features that can be reliably used to distinguish the cell groups. The hierarchy Census uses is computationally efficient to create, but in principle, any cellular ontology and corresponding marker gene-sets could be specified by a user for the analysis. Nonetheless, as the manuscript demonstrates, the hierarchy used by Census works exceptionally well for the task of cell type annotation.

**Figure 2.**
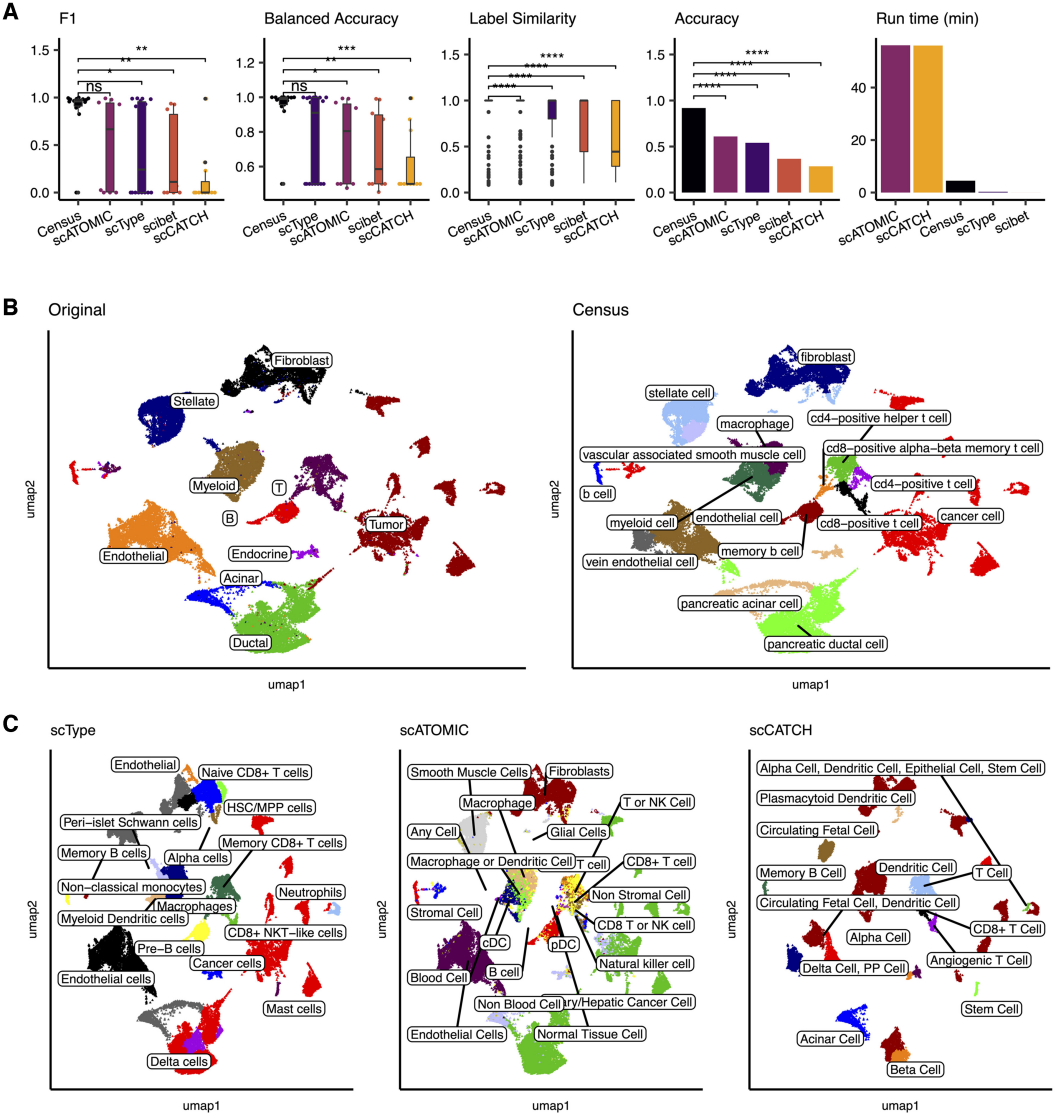
Benchmarking Census on a pancreatic cancer dataset. (A) Benchmarking Census against four other automated cell-type annotation methods using a pancreatic cancer dataset. Boxplots show median (line), 25th and 75th percentiles (box) and 1.5xIQR (whiskers). Points represent outliers; Wilcoxon tests, **** *P* < 1e−4; *** *P* < 1e−3; ** *P* < 1e−2, * *P* < 0.05; ns, not significant. (B) Uniform manifold approximation and projection (UMAP) plots of 57 530 cells from a pancreatic cancer study colored by cell-type with overlaid labels. Left, original study annotations; right, Census annotations. (C) UMAP plots of the same study in (B) annotated using scType (left), scATOMIC (middle), and scCATCH (right). HSC/MPP, hematopoietic/multipotent progenitor.

We first benchmarked Census against four other state-of-the-art automated annotation methods [scType (Ianevski *et al.* 2022), scATOMIC (Nofech-Mozes *et al.* 2022), scibet (Li *et al.* 2020), scCATCH (Shao *et al.* 2020)] using a pancreatic cancer dataset (Peng *et al.* 2019) that included 57 530 normal and malignant epithelial cells as well as nonmalignant stromal and immune cells. Annotation performance was evaluated by five metrics: F1 score, balanced accuracy, total accuracy, run time, and “label similarity” scores that we computed using our predefined cell hierarchy to quantify closeness of the predicted label to the study’s original annotation (see Methods). Census was the top performing method with regards to prediction quality, where it had a higher mean F1 score and balanced accuracy than the second-place method and significantly higher scores than the others (Wilcoxon, *P* < 0.05), and it had significantly higher label similarity and accuracy than all methods (Wilcoxon, *P* < 2e−16, Fig. 2A). While Census was not the fastest method, it ran in 4.5 min (range: 1 s to 56 min, Fig. 2A), correctly identified 9/10 major cell-types, distinguished between normal and malignant epithelial cells, and identified deeper immune subtypes than originally annotated (Fig. 2B, Supplementary Fig. S2A). Lastly, because the label similarity score used the cell-hierarchy on which Census was trained, we also evaluated label similarity using a recently published atlas of human cell ontology (Chen *et al.* 2022). We counted the number of cells that were annotated correctly or within the same lineage as the original author annotation. For example, if the true annotation for a given cell was “CD4 T cell” and a method called it “T cell” this would be counted positively as CD4 T cells are a direct descendent of the general T cell lineage in hECA (Supplementary Fig. S1). However, if a method called the cell a “CD8 T cell” or a “B cell”, these are directly within the parent or child lineages of “CD4 T cell” in hECA, and thus this cell would not be counted. Census annotated at least 1.75 times or more cells correctly or within the same lineage as the correct annotation than all other methods tested (Supplementary Fig. S2B).

**Figure 3.**
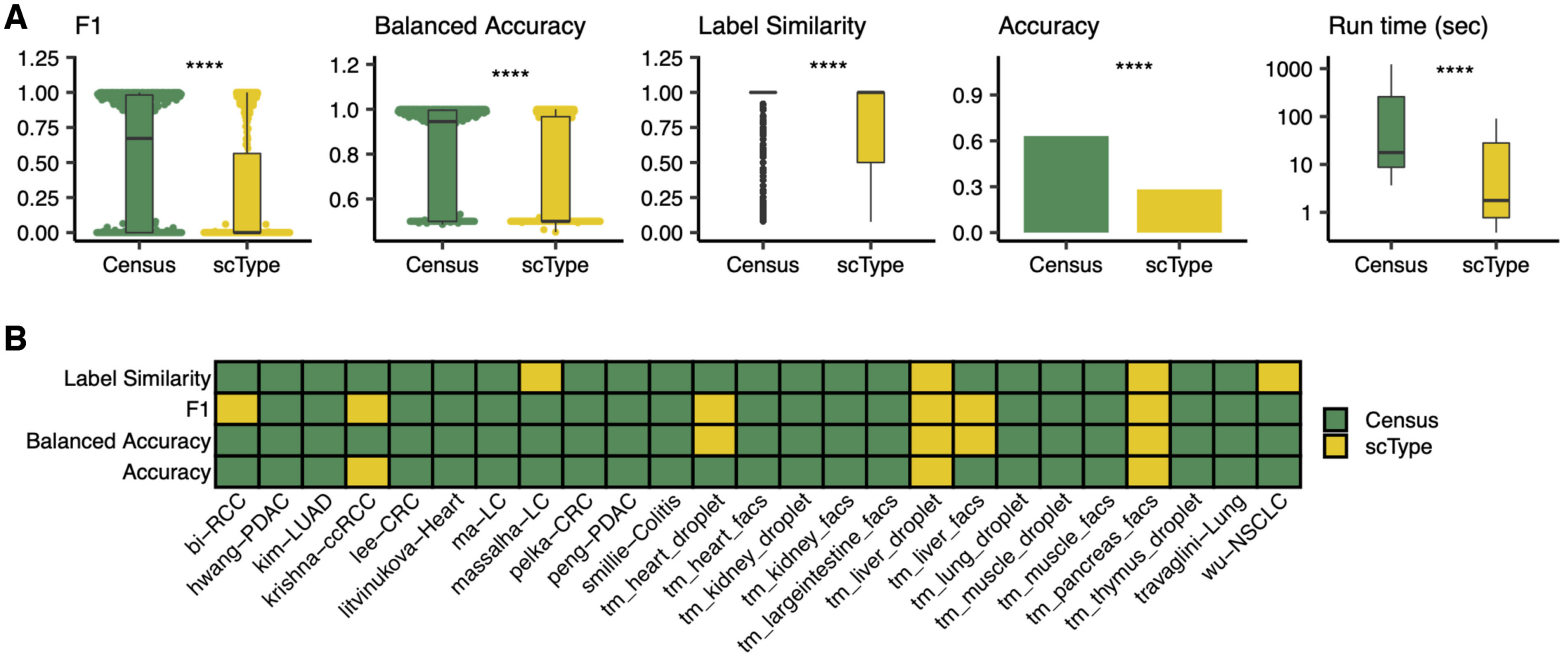
Summary statistics of extended benchmarking of Census on normal and cancer, human and mouse tissues. (**A**) Performance metrics comparing Census and scType on 25 tissues. Label similarity is a computed metric quantifying closeness of the predicted versus author annotated cell annotation on the Census Tabula Sapiens cell-type hierarchy from Fig. 1. Boxplots show median (line), 25th and 75th percentiles (box), and 1.5xIQR (whiskers). Points represent outliers; Wilcoxon tests, **** P < le−4. (**B**) Heatmap showing the top performing method across four evaluation metrics for 25 commonly evaluable tissues. tm, Tabula Muris.

In terms of accuracy, speed, and precision, Census and scType were the top two methods (Fig. 2B and C). We thus proceeded to assess their annotation performance on 44 other challenging normal and cancer datasets from human tissues and from the Tabula Muris (Schaum *et al.* 2018); these data included 1 769 071 total cells from 105 harmonized cell labels—all from new datasets not seen by the model during training and testing. In aggregate, Census had significantly higher F1 scores, balanced accuracies, label similarities, and overall accuracies than scType (all Wilcoxon *P* < 2e−16, Fig. 3A), although scType had shorter run times (Wilcoxon *P* < 2e−16, Fig. 3A) – though Census was still very fast with an average annotation speed of 13 000 cells/min. Looking at prediction performance in individual studies, Census had higher mean values than scType in 83/100 commonly evaluable metrics (Fig. 3B). These data place Census as a top automated annotation method (Supplementary Table S1).

**Figure 4.**
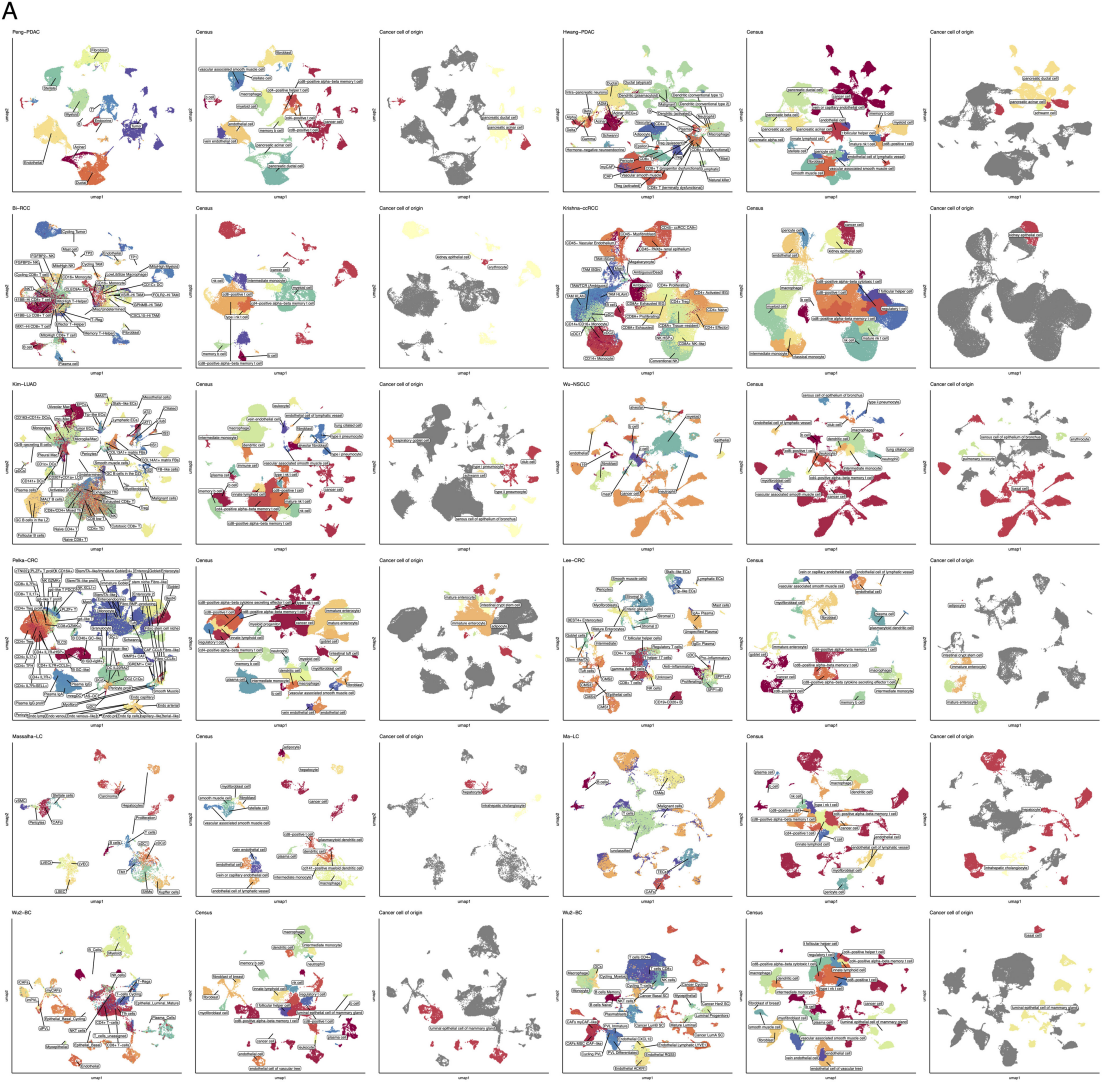
Census identifies normal, malignant, and cancer cells of origin. (A) Uniform manifold approximation and projection (UMAP) plots of 12 cancer datasets colored by cell-type annotation with overlaid labels. Each row plots data from two studies from one tissue type, the left three plots are from one study and right three from the second. From each set of three plots: left, original author annotations; middle, Census annotation; right, Census predicted cancer cell of origin. PDAC, pancreatic ductal adenocarcinoma; RCC, renal cell carcinoma; ccRCC, clear cell renal cell carcinoma; LUAD, lung adenocarcinoma; NSCLC, non-small cell lung cancer; CRC, colorectal cancer; LC, liver cancer; BC, breast cancer. Cell-type abbreviations: ADM, acinar-ductal metaplasia; CAF: cancer-associated fibroblast; myCAF: cancer-associated myofibroblast; Treg, regulatory T-cell; TP, tumor; TAM, tumor-associated macrophage; NK, natural killer; pDC, plasmacytoid dendritic cell; cDC, conventional dendritic cell; Mac, macrophage; GC, germinal center; EC, endothelial cell; FB-fibroblast; TS, tumor cell; AT1/2, alveolar type 1–2; LVEC, liver vascular endothelial cell; LSEC, liver sinusoidal endothelial cell; SAM, scar-associated macrophage. vSMC, vascular smooth muscle cell; TM, tumor-associated macrophage; TEC, tumor epithelial cell; imPVL, immature perivascular like; dpPVL, differentiated PVL.

**Figure 5.**
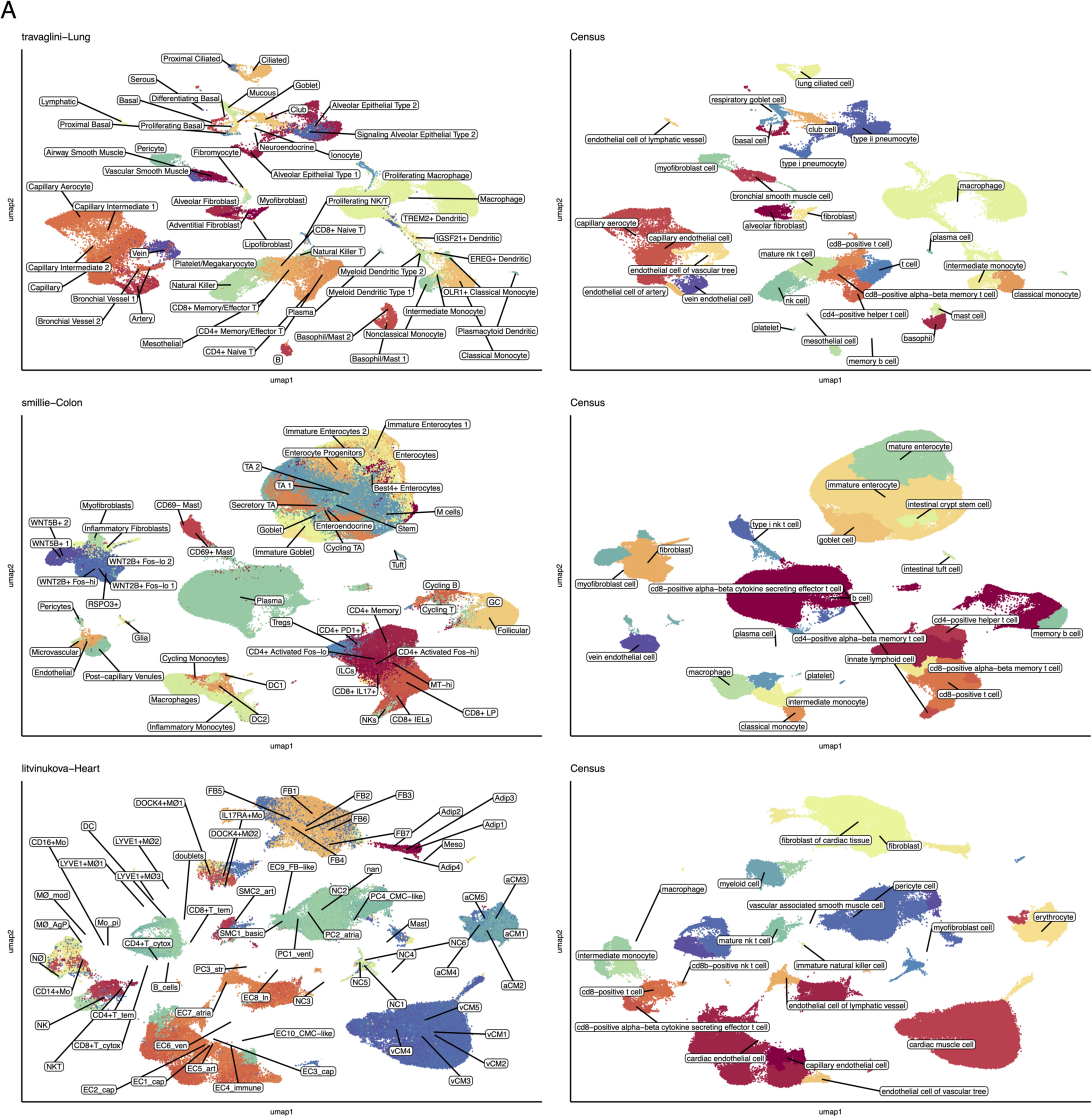
Evaluating Census on non-cancer, deeply annotated human tissue. (A) Uniform manifold approximation and projection (UMAP) plots of three datasets colored by cell-type annotation with overlaid labels. Each row plots data from one tissue type. Top, lung; middle, colon; bottom, heart. Left column annotated by the original author annotations, right by Census annotation. NK, natural killer; TA, transit amplifying; Treg, regulatory T-cell; GC, germinal center B-cell; ILC, innate lymphoid cell; DC, dendritic cell; FB, fibroblast; Adip, adipocyte; SMC, smooth muscle cell; Mo, macrophage; EC, endothelial cell; PC, pericyte cell; NC, neuronal cell; aCM, atrial muscle cell; vCM, ventricular muscle cell.

**Figure 6.**
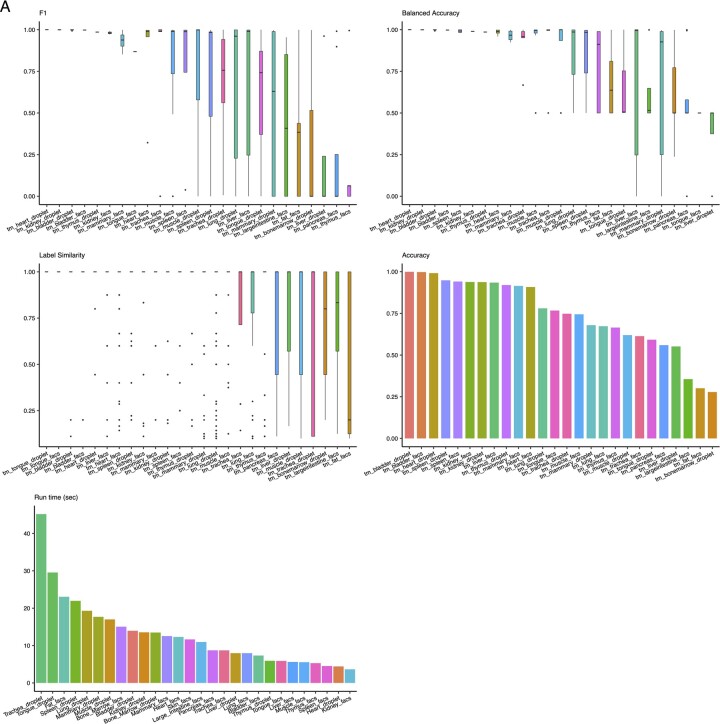
Benchmarking Census on the Tabula Moris. (A) Evaluating Census prediction performance on tissues from the Tabula Muris. Label similarity is a computed metric quantifying closeness of the predicted versus author annotated cell annotation on the Census Tabula Sapiens cell-type hierarchy from Fig. 1C. Boxplots show median (line), 25th and 75th percentiles (box), and l.5xIQR (whiskers). Points represent outliers.

Inspection of specific results highlights the power of Census in diverse settings. In two datasets each of human breast (Wu *et al.* 2020, Wu *et al.* 2021b), colon (Lee *et al.* 2020, Pelka *et al.* 2021), kidney (Bi *et al.* 2021, Krishna *et al.* 2021), liver (Massalha *et al.* 2020, Ma *et al.* 2021), lung (Kim *et al.* 2020, Wu *et al.* 2021a), and pancreas (Peng *et al.* 2019, Hwang *et al.* 2022) cancers, Census identified malignant cells and distinguished them from concurrent normal epithelium (Fig. 4A). It also identified the likely cell of origin for cancer cells. For example, in liver cancer, Census correctly distinguished between known hepatocellular versus cholangiocarcinoma cells (Fig. 4A), consistent with the original study’s clinical annotations (Supplementary Fig. S2B). In pancreatic ductal adenocarcinoma it identified most malignant cells as ductal cell type-origin and a few tumors as having transcriptional states similar to acinar cell-types, and in colon cancers the cells of origin were from the enterocyte lineage (Fig. 4A). On deeply annotated normal tissue atlases, Census distinguished between several cell subtypes. For example, it identified aerocytes and capillary, vein, artery, and lymphatic endothelial cells, distinguished between alveolar, adventitial, and myo-fibroblasts, and identified several T-cell and myeloid cell subsets in lung (Travaglini *et al.* 2020), colon (Smillie *et al.* 2019), and heart (Litviňuková *et al.* 2020) tissues (Fig. 5A). Census performance is not human-specific, and it also had excellent performance on mouse tissues when tested on droplet and plate-based sequencing samples from the Tabula Muris (Schaum *et al.* 2018) (Fig. 6A), with a mean balanced accuracy of 0.8, label similarity of 0.89, and run time of 13 s across all tissues. Overall, Census correctly identified 81/105 tested cell subtypes (compared to 35/89 by scType), and Census’s prediction accuracy per cell-type correlated with the corresponding number of cells used for model training (Spearman *ρ* = 0.26, *P* = 0.01).

**Figure 7.**
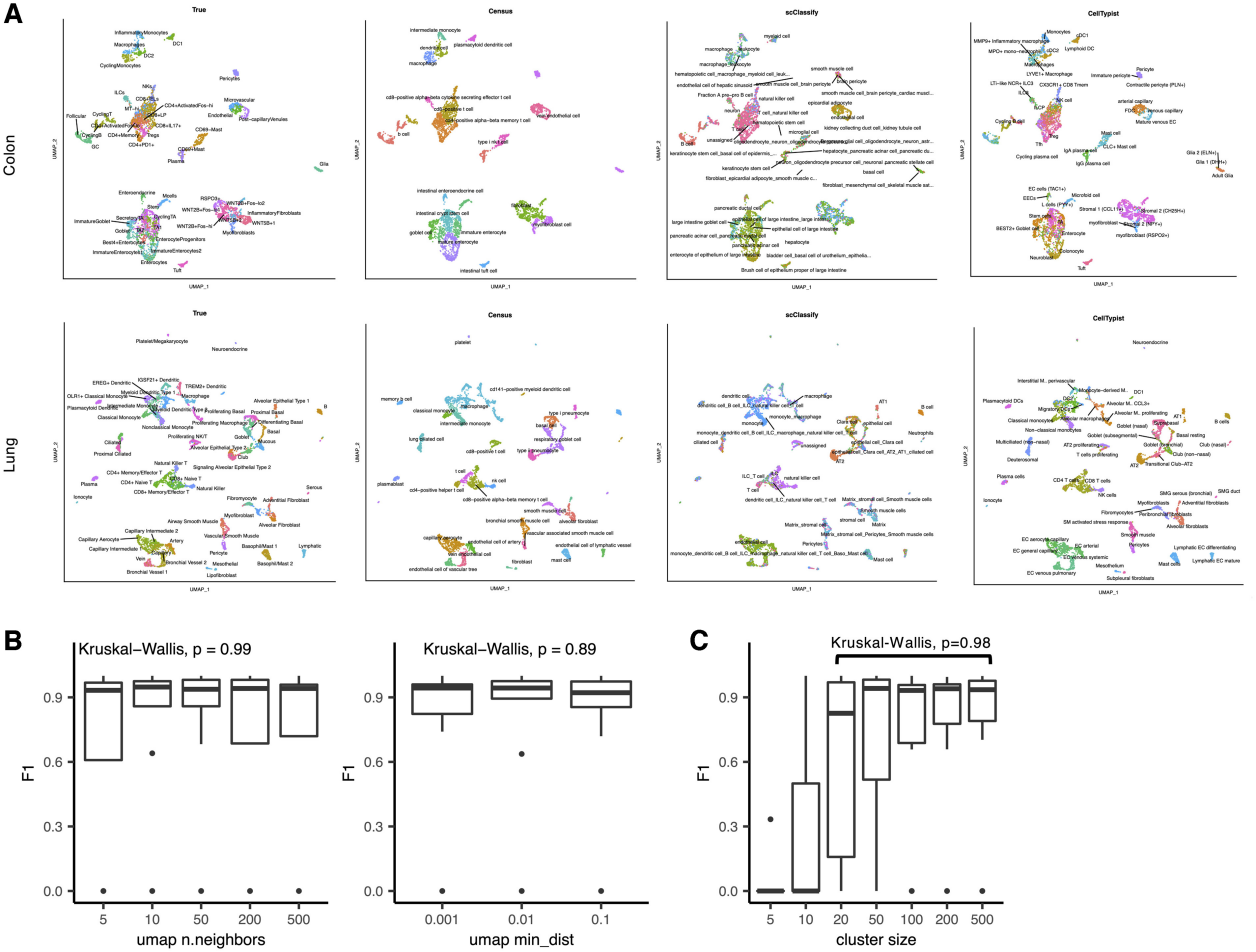
Analysis of performance with varying UMAP hyperparameters. (A) Uniform manifold approximation and projection (UMAP) plots of two datasets colored by cell-type annotation with overlaid labels. Census predictions are compared to original author annotations, scClassify, and CellTypist. (B) Boxplots of cell-type F1 scores for predicting the Peng *et al.* pancreatic cancer dataset when the UMAP “n.neighbors” parameter was varied (left) and when the “min_dist” hyperparameter was varied (right). Boxplots show median (line), 25th and 75th percentiles (box), and 1.5x1QR (whiskers). Points represent outliers. (C) Boxplots of cell-type F1 scores for the same dataset in (B) when cell-types were down-sampled.

The aforementioned analyses collectively demonstrate Census’s excellent performance under a diverse set of challenging tests. We next performed 3 additional benchmarking experiments or comparisons. In the previous analyses (Figs 2–6), we compared Census against fully automated annotation methods that could identify tumor cells. We next compared Census against two other methods that do not do fully automated cancer cell identification or cell of origin prediction but which share methodological similarities to Census. The methods selected were scClassify (Lin *et al.* 2020), which constructs its own cell hierarchy and uses ensemble learning to automate cell-type annotation, and CellTypist (Domínguez Conde *et al.* 2023), a new tool with demonstrated automated annotation performance that has multi-label and majority voting functionality which addresses the same problem as the label stabilizing algorithm in Census. We used pretrained models from these methods and Census to annotate the lung (Travaglini *et al.* 2020) and colon (Smillie *et al.* 2019) cell atlases analyzed in Fig. 5 (scClassify and CellTypist did not include pretrained heart models). This analysis and examination of the annotation results reveal the strengths of Census (Fig. 7A). First, Census benefits from a larger selection of organ types and can annotate more cell-types than all methods compared with in this manuscript. Second, Census has multiple options for selecting the organ(s) from which to annotate cells. This eliminates impossible annotations that other methods may choose (i.e. cell-types specific to non-present organs) and increases Census’s annotation accuracy. Third, the core Census model can identify cancer cells and naturally predicts their predicted cell of origin. And like the other methods, Census can seamlessly be trained on new data for customized applications.

We next investigated the effect of specific parameters on Census’s performance. Census uses a UMAP embedding to refine cell-type annotations, and changes in cell proportions or UMAP hyperparameters could potentially influence the final annotations. Using the pancreatic cancer dataset analyzed in Fig. 2, we recomputed the UMAP embedding while varying two parameters: “n.neighbors”, which determines the number of neighboring points used for approximating the manifold, and “min.dist”, which controls how tightly the points are compressed together in the final embedding. We annotated the each iteration with Census and compared the cell-type F1 scores across all iterations and found no difference with varying either parameter (n.neighbors Kruskal-Wallis *P* = 0.99; min.dist Kruskal-Wallis *P* = 0.89, Fig. 7B). UMAP clustering can also be affected by the number of cells per cluster. For the same dataset, we down-sampled the number of cells from 5 to 500 in each cluster, recomputed the UMAP embedding and annotated the data with Census. When 20 or more cells were present for a cell-type, there was no significant difference in prediction F1 scores (Kruskal-Wallis *P* = 0.98, Fig. 7C). However, performance decreased when there were fewer than 20 cells/cell-type. These analyses demonstrate that Census performance is robust with respect to changes in sample size (at >20 cells/cell-type) and UMAP hyperparameters.

## 4 Discussion

In summary, Census enables easy and fast, fully automated cell-type identification from scRNA-seq data using a hierarchical cell-type reference. It significantly outperforms other state-of-the-art methods when extensively tested on human and mouse, cancer and normal tissues. Utilization of a cell-type hierarchy provides a natural interpretation of annotation results and aids in Census’s superior performance. It also allows cell type identification at different resolutions, which can be advantageous when comparing results from different datasets, across species, and in the context of complex diseases such as cancer. While the core Census model is trained on the Tabula Sapiens (Jones *et al.* 2022), with one line of code users can seamlessly train their own models with other references for customized applications.

The two key features underlying Census’s superior performance are the single-cell hierarchical classification framework followed by the cluster label stabilizing algorithm in UMAP space, which pools information from local cell neighborhoods to identify the most likely label. It has previously been shown that UMAP does not optimally preserve either global or local structure, and that there is a high degree of variability in possible 2D-embeddings that can be obtained from typical scRNA-seq data (Chari and Pachter 2022). However, we found that in practice, in large datasets very closely clustered cells in UMAP typically are the same cell-type even when cell-type clustering is standardly done in principle component space (Supplementary Fig. S3), and varying UMAP hyperparameters within recommended limits does not significantly change the final embedding or Census predictions. Thus, the label-stabilizing algorithm used by Census thus takes advantage of the observation that extremely closely clustered cells in UMAP space in large datasets are usually of the same cell-type and this improves the final prediction results. However, to mitigate suboptimal UMAP structure preservation, the label-stabilizing algorithm finely clusters the data such that there are typically 10× more clusters than final observed cell-types. Our analysis of clustering performance in UMAP and principle component space suggested that although these two approaches usually result in very similar clusters, some discrepancies do occur and lead to very distant cells in UMAP belonging to the same principle component cluster. During our model development, this negatively impacted the label-stabilizing algorithm and overall prediction metrics. Thus, despite limitations of UMAP, we chose to cluster in UMAP space instead of principle component space as it demonstrated the best overall results as well as significant improvements in prediction over comparable methods.

While Census is fast and has excellent performance across a range of tissues and disease states, we note some limitations of the method. First, Census does not identify unknown or new cell-types; all cell-types in the test dataset should be included in the training data. However, Census does report the classification probability at each level of the cell-type hierarchy for each cellular barcode. Users can compare these probabilities to identify anomalies to identify cells with inadequate classification confidence; a default threshold is not included as we found that this would be dataset-specific. Second, the cell-type specific performance of Census correlated with the number of cells used for training; it is expected that for rarer cell-types with sparse data the performance will be weaker. Users should take caution when annotating cells without sufficient training data, and performance will be improved if data from multiple independent studies can be used for training. Third, Census uses the UMAP embedding to refine labels. While this feature improves annotation overall, Census will be unable to distinguish two cell-types that are overlapping in UMAP space in very large datasets. In such scenarios, this can be mitigated by subsetting the data and creating separate embeddings for different classes of cells, e.g. separate UMAPs for immune versus epithelial cells. Fourth, the core Census model trained on the Tabula Sapiens and CCLE can identify malignant epithelial cells for select organs, users may train their own custom models using relevant data for these organs, or for identifying other cancer types such as sarcomas or lymphomas. Lastly, in this manuscript one of the metrics used to assess prediction performance was the “label similarity score”, i.e. the distance in the Census hierarchical tree from the predicted label to the ground truth label. These scores may favor Census predictions because the algorithm explicitly follows this hierarchy, whereas other methods may not predict all the same labels. This warrants some caution in interpreting those scores across methods.

Nonetheless Census provides an easy and fast way to quickly annotate cells in the majority of human samples with high resolution and with an associated cell-type hierarchy that allows users to identify related cell-types or dataset specific appropriate label resolutions. Census is available on our Github: https://github.com/sjdlabgroup/Census.

## Supplementary Material

btad714_Supplementary_Data

## Data Availability

Census is available on our Github: https://github.com/sjdlabgroup/Census and at Zenodo: https://zenodo.org/records/7017103. All data and code to reproduce the analyses and figures are available in the same Census Github repository and from the corresponding author upon request.
