# Supplementary material for: Hierarchical and automated cell-type annotation and inference of cancer cell of origin with Census
Source: Bioinformatics. 2023 Nov 27;39(12):btad714. doi: 10.1093/bioinformatics/btad714 (PMC10713118; doi:10.1093/bioinformatics/btad714)
Supplement: btad714_Supplementary_Data [file btad714_supplementary_data.zip › Supplemental Information.pdf]

Figure S1

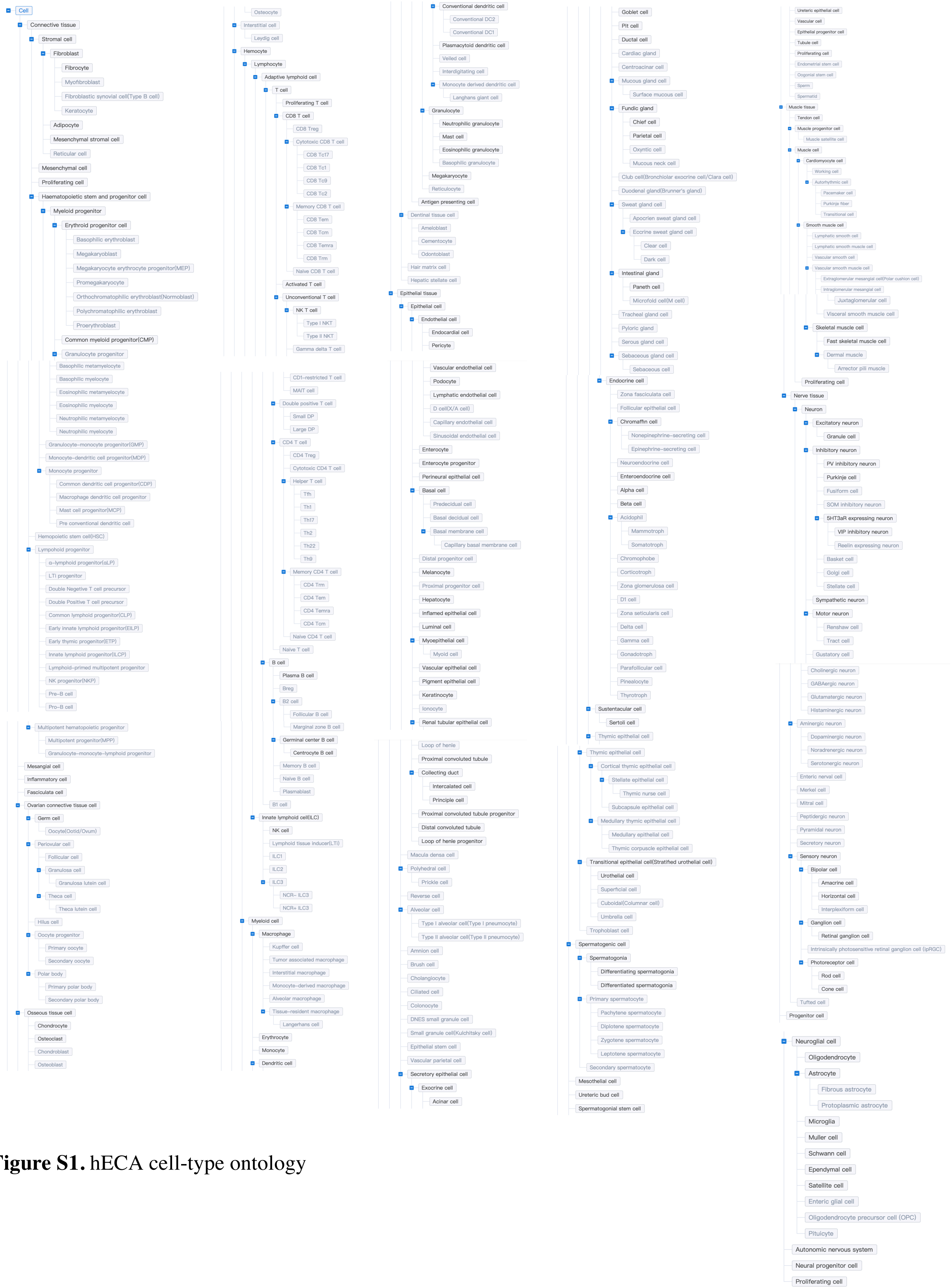

Figure S1. hECA cell-type ontology

Figure S2

A

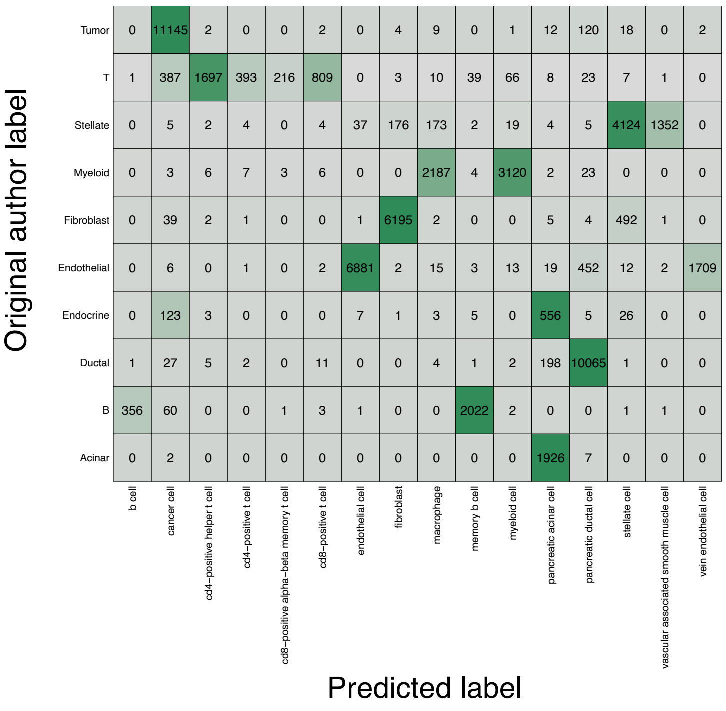

B

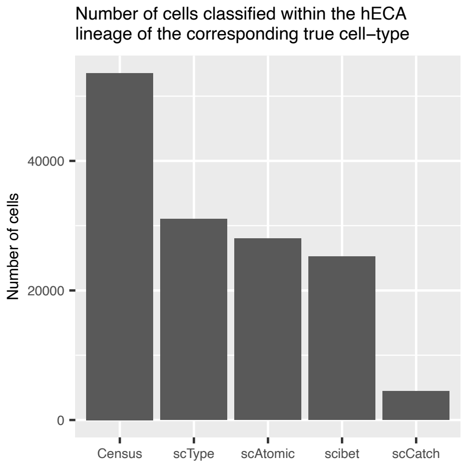

C

True cancer type

Predicted cell of origin

	Intrahepatic cholangiocarcinoma	Hepatocellular carcinoma
Intrahepatic cholangiocyte	2390	297
hepatocyte	21	18299

Figure S2. (A) Heatmap showing the correspondence between predicted labels vs. the original author labels for the Peng et al. pancreatic cancer dataset. **(B)** Bar-plot showing the number of cells Census and the other methods annotated correctly or within the same lineage as the correct annotation using the hECA cell ontology from Fig. S1. **(C)** Table showing the correspondence between Census cancer cell of origin predictions vs. the clinically identified cancer types for the Ma et al. liver cancer study.

Figure S3

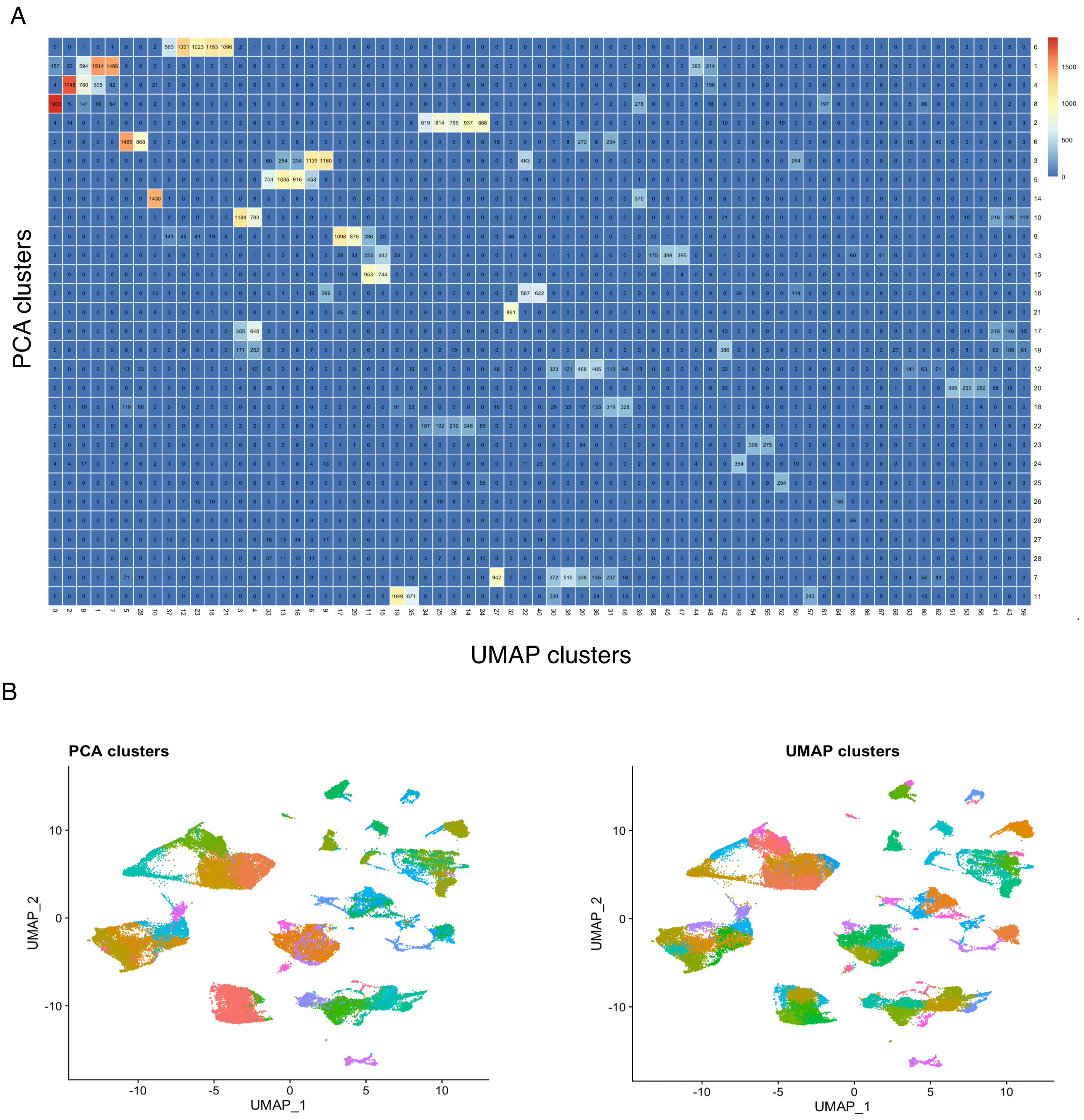

Figure S3. (A) Comparing cell clustering in PCA vs. UMAP space for the pancreatic cancer dataset. The values in the heatmap indicate the number of cells that in the same cluster in UMAP and PCA space. This heatmap shows that practical clustering results are largely consistent in the two spaces. **(B)** UMAP plots colored by either PCA or UMAP derived clusters showing the similar cluster assignments in both methods.
